# Protect the Little Boy

**Published:** 1913-08

**Authors:** 


### Protect the Little Boy

We commend to individuals and societies especially interested in conserving the morals of the nation, a factor often overlooked—the innocence and purity of the little boy. We are not attempting to upset the world-wide and world-old notion that sexual morality and respectability are feminine attributes, nor the converse proposition that sexual obloquy attaches particularly to that sex. We do not believe, on abstract principle, that such should be the case, but we fear that any tendency to the practical establishment of equality of the sexes in this respect will tend to level downward, not upward.

But, in everything but anatomy and social potentialities, a little child is sexless. We can see no logic in the explicit or tacit contention that the boy's innocence, virtue and purity, are different from the same attributes of the girl.

Some years ago, half a dozen boys and young men, 18 to 21 years old, of supposedly good families, at least the leading families of a small town, cajoled a little girl of five, of a similar family, into accompanying them to the outskirts of the town. In a deserted house they stripped her and subjected her to all sorts of indignities. What was the result? Some of our readers will answer legal punishment and imprisonment, others tarring and feathering, others, thinking of lower latitudes and, possibly of differences of race, will suggest a hunt with blood hounds and burning at the stake. These are natural suggestions, but they are wrong, because we deliberately mis-stated the case. The truth is that it was a band of girls and young ladies who victimized a little boy. And the result was that the parents were highly indignant, that most of the people laughed at the episode, that some of the girls were ashamed of what they had done and that the others lost more or less in the esteem of their neighbors.

We don't know the future career of this boy, but we do know that two of the worst rakes of our acquaintance were seduced when they were about fourteen by mature women responsible for their intellectual and moral training. These two boys have collected the damages due them a hundred times over, but from girls in no way responsible personally.

If society and the law can see no abstract principle which demands the protection of childish innocence, regardless of sex, there is, nevertheless, the reflex action on feminine virtue to be considered.

